# Processing of different word list lengths during encoding and retrieval in Broca’s area

**DOI:** 10.3389/fnhum.2026.1757160

**Published:** 2026-05-29

**Authors:** Viktors Veliks, Aleksandrs Kolesovs, Juris Porozovs, Dmitrijs Igonins

**Affiliations:** 1 Faculty of Medicine and Lifesciences, Institute of Clinical and Preventive Medicine, University of Latvia, Riga, Latvia; 2 Faculty of Medicine and Lifesciences, University of Latvia, Riga, Latvia; 3 Faculty of Education Sciences and Psychology, University of Latvia, Riga, Latvia

**Keywords:** encoding, episodic memory, recognition, spectral power, time frequency, word list length

## Abstract

Broca’s area is a region of the brain involved in the processing of verbal information, including memory encoding and retrieval. This study examined differences in the neural correlates of encoding and retrieval in Broca’s area using word lists of varying lengths. Differences in the encoding and retrieval processes were assessed using word lists of different lengths: short (2–29), medium (30–59), and long (60–225) Latvian language nouns. In total, 23 participants completed the memory task. Each participant performed memory tasks with two short lists, two medium lists, and one long list, with varying list lengths within each diapason. We considered the activity of the F3 and F7 to represent our region of interest. We compared time-frequency (TF) data from encoding and retrieval across list lengths. The results revealed significant differences in TF plots for encoding and for correct and incorrect retrieval of information up to a list length of 50 words. Further increases in list length indicated greater similarity in brain functional patterns. For correct stimulus recognition or rejection of a distractor, the observed differences in TF depended on list length, and these differences were more pronounced under correct stimulus recognition. Encoding and correct answers during retrieval also showed statistically significant differences from incorrect answers (misses or false alerts) at different list lengths. Spectral power changes exhibited a nonlinear shape during both encoding and retrieval. The possible mechanisms differed during encoding and correct recognition versus mistakes.

## 1 Introduction

The role of Broca’s area in language and verbal information processing remains a topic of analysis (Bulut, 2023; Tremblay and Dick, 2016). The area’s involvement in the network of verbal and acoustic processing (Chai et al., 2018) links its activity to memory processes, which are crucial to human functioning. Despite the fact that memory has been studied from different perspectives (Chai et al., 2018; Hill and Dunbar, 2003; Peters et al., 2022; Tromp et al., 2015), little is known about its functioning under high memory loads or the neural differences between information accumulation and storage in accessible conditions. The majority of studies address these issues in the context of working memory (Baddeley, 2012), focusing on oscillatory activity and memory load (the number of items to be memorized); however, the EEG spectral correlates of encoding, storage, and retrieval under loads greater than 9–25 items have been less investigated (Cary and Reder, 2003; Dennis et al., 2008). To expand on current knowledge of the neurophysiological mechanisms underlying memory capacity limits, the present study examined neurophysiological markers of memory processing during the encoding and recognition of verbal information (word lists) in Broca’s area under highly variable memory loads (2–225 words).

Whether list length (set size) affects recognition accuracy and speed remains unresolved (Dennis et al., 2008). The List Length Effect (LLE) shows that shorter lists yield higher hit rates and lower false alarm rates (Ratcliff and Murdock, 1976; Murnane and Shiffrin, 1991; Cary and Reder, 2003). At the same time, some studies have reported a “zero” LLE (Turner et al., 2013; Dennis et al., 2008). These results have challenged recognition memory models and have prompted debate over whether the LLE is explained by differences in list length or storage interval (Annis et al., 2015; Murdock and Kahana, 1993; Dennis and Humphreys, 2001).

By testing several primary variables that potentially influence the LLE, such as storage interval, the number of items between an item’s occurrence in the list and its test, and serial item position in the list, and by manipulating list length, Ohrt and Gronlund (1999) validated the LLE in a recognition task. Despite the debate over the status of the LLE and its potential to verify cognitive models of recognition memory, the effect has sufficiently strong empirical validity and requires further study across longer lists. This is supported by a change in the function describing the dependence of the information retrieval rate on memory. Early evidence for this change came from Burrows and Okada (1975), whose study found that recognition reaction times were linear up to lists of 6 items and logarithmic thereafter, up to 25 items. This change may reflect sequential information retrieval in short-term (working) memory, as first demonstrated by Sternberg (1966), along with a combination of both sequential and parallel processing in long-term memory (LTM).

This may be evidence of differences in the cognitive processes and mechanisms involved in encoding and retrieving information in episodic memory with increasing storage capacity. These discoveries require further study of the corresponding mechanisms of information processing, in addition to the development of theoretical models of them (Graboi and Lisman, 2003). Existing LLE studies have typically used stimulus sets of up to 100 items and compared LLE values at different list lengths (Cary and Reder, 2003; Dennis et al., 2008).

Statements about the logarithmic function approximating the relationship between list length and memory retrieval rate have been made primarily within these limits and are based on a limited number of empirical points. Thus, they have approximated their empirical data with a logarithmic function over four points corresponding to set sizes of 24, 48, 72, and 96 items (words). Judging from a previous study (Peters et al., 2022), the logarithmic fit is a rough approximation of the empirical data, and its divergence from the data may increase as the set size and number of points increase. As Burrows and Okada (1975) noted, the logarithmic fit was also a crude approximation, failing to capture a clear nonlinear effect in the 7–12 list length interval. Extensive verbal memory studies are very important for assessing human verbal memory capacity and clarifying the peculiarities of information encoding. Combining research on the storage of a long list of information in memory with the registration of brain bioelectrical activity enables a more precise understanding of the brain’s peculiarities in information storage.

Conceptualizing these schemas as discrete units allows their use to estimate the potential limits of memory capacity, particularly in the domain of episodic social memory. Existing accounts suggest a gradual reduction in episodic specificity as the size of social networks approaches cognitive limits, implying an increasing contribution of semantic memory representations (Sutcliffe et al., 2012).

It should be noted that the present study focuses on verbal episodic memory, systematically manipulating the number of encoded units (words), which have substantially lower informational density than social memory schemas. Rather than comparing encoding mechanisms across episodic and semantic domains, we aim to identify neurophysiological markers of memory processing under a wide range of memory loads. Specifically, we examine oscillatory brain activity during encoding and retrieval as a function of the number of items to be memorized.

Our expectation that the modulation of neurophysiological correlates of memory is based on findings regarding oscillatory power in multiple EEG frequency bands (alpha, beta, and gamma) and on the observation of burst-like events in power analyses (Günseli et al., 2026; Kavanaugh et al., 2026; Omelyusik et al., 2026). Modulations of theta frequency bands were also revealed in studies on information encoding, processing, and following decision-making (Cavanah and Fiebelkorn, 2026; Chmiel and Kurpas, 2026; Wynn et al., 2024). In addition, the continuous analysis of the effects of synchronization and desynchronization of neurophysiological activity on memory processes (Hanslmayr et al., 2009, 2016; Rao et al., 2025; Solomon et al., 2017) allows us to expect variability in oscillatory power during encoding and during correct and incorrect retrieval.

Accounting for Broca’s area’s involvement in verbal working memory, phonological processing, and articulatory rehearsal, it could serve as a region that accumulates the increasing cognitive load associated with longer word lists. As the list length grows, Broca’s area is expected to demonstrate altered oscillatory activity, reflecting a shift from short-term sequential retrieval to more distributed long-term memory processing. A pilot study (Veliks et al., 2025) has revealed changes in EEG wave spectrum power and brain wave interaction dynamics across different list lengths during the encoding of information in memory. Therefore, the present study examines whether and how the length of word lists modulates neural activity in Broca’s area during both encoding and recognition.

## 2 Methods

### 2.1 Participants

The study included data from 23 female student volunteers in the pedagogy program at the Riga Teacher Training and Educational Management Academy, aged 38 ± 8 years. All participants were right-handed native Latvian speakers without vision or cognitive impairments. The higher efficacy of women on verbal memory tasks (e.g., Wong et al., 2020) and the composition of the teachers’ population in Latvia, with over 80% women, determined the formation of a homogeneous sample based on participant gender. The research was conducted in accordance with the national legislation of the Republic of Latvia and the Declaration of Helsinki. As part of a scientific cooperation, the current study used anonymized data from a previous project.

### 2.2 Procedure

The study followed a within-subjects design with a positionally balanced presentation of medium (M) and short (S) word lists in the first session and an additional session involving long (L) words. Therefore, each participant completed five memory tasks following a sequence of M–S–S–M–L. List lengths were balanced according to the number of words for each participant.

Word stimuli in the present study were nouns selected from among the 1,500 most common words in spoken Latvian. Concrete and abstract nouns of different lengths (from 3 to 14 letters, mean ± SD was 6.58 ± 1.89) were used in this study. Compound nouns were not included. Word examples: “Kartupelis” (Potato), “Lācis” (Bear), “Zvaigzne” (Star), “Doma” (Thought), “Darbība” (Action), “Gadījums” (Chance). For the creation of experimental lists, the list lengths were chosen, and words were selected from the main list.

Five tasks of unique and different lengths were prepared for each participant and divided into two separate sessions (encoding and retrieval). Words were semi-automatically selected using a Matlab script for randomization and for the formation of the following tasks: two tasks with a random list length of 2–29 words (short), two tasks with a random list length of 30–59 words (medium), and one task with a random list length of 60–225 words (long). Participants were not required to use any mnemonic techniques to memorize the words.

Word characteristics were balanced by a random selection of words from a permuted list of the most common words. Words that had already been selected were not included in the lists again. Therefore, each participant had their own combination and sequence of non-repetitive words in the tasks.

Each recognition test included half of the randomly selected words from the encoding list, which were supplemented by the corresponding number of randomly selected distractors from a total set of 1,500 words. Each distractor appeared in the tests offered to a participant only once. The randomization and permutation of words, combined with the integration of the obtained data across the sample, provided additional control over linguistic variables (e.g., word length, number of syllables, word frequency, and so on), the possible effects of semantic clustering during memorization, and the effects of serial position in encoding lists across participants.

Participants’ attentional control methods were not used during the encoding task, given evidence that neural representations of high-frequency words are automatically activated when words are presented, even when they are not the primary focus of attention (Alexandrov et al., 2011; Shtyrov and Pulvermüller, 2007). The recognition test was presented without delay after the completion of the list coding phase. The participants’ reading of the standard test instructions was considered a filler task that blocked the possible repetition of recently presented units in working memory and eliminated the effect of recency in recognition tasks.

The experiment was performed in a quiet room. During the recording, participants sat in a comfortable position on a chair. During the experiment, the light was turned off.

Psychtoolbox-3 software^1^ was used to present the words (Kleiner et al., 2007). Black-colored words (in Arial font and with a size of 50 pixels) were presented on a white screen. The stimulus words were presented on a 17” LCD monitor with a resolution of 1,200 × 1,024, and the participants were seated approximately 100 cm away from it.

**Figure 1:**
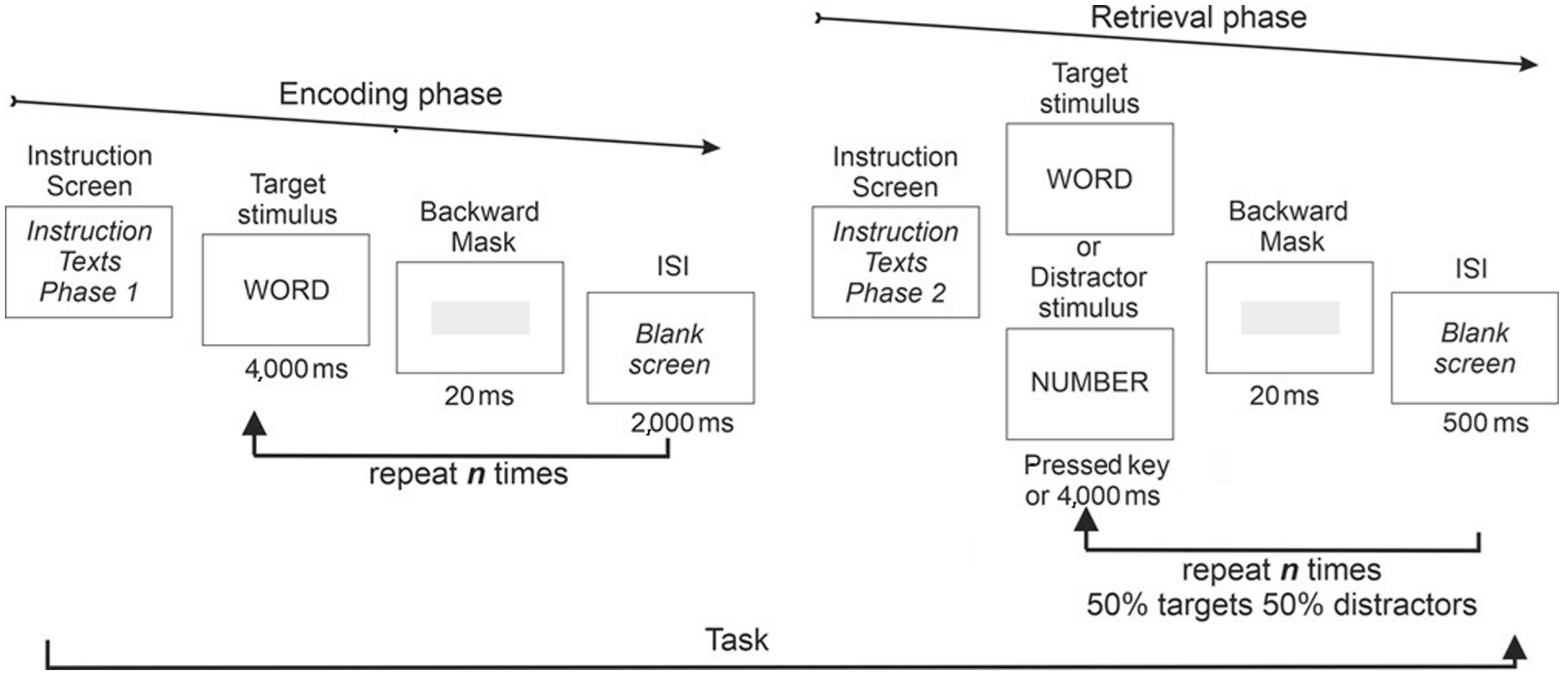
Diagram of the experiment.

During the encoding task, the exposure time for each stimulus word was 4,000 ms with an interstimulus interval (ISI) of 2,000 ms (Figure 1). During the retrieval task, words were presented until participants pressed the “Z” key on the keyboard for a new word or the “2” key if the word was presented in the encoding list or after 4,000 ms if the participants did not press any key. After that, an ISI of 500 ms followed, after which the next word was presented.

The participants’ readiness to perform the retrieval tasks was signaled by the pressing of the “space” bar. In each subsequent trial of the recognition test, participants were required to determine as quickly as possible (and as accurately as possible) whether the presented word appeared in the encoding list. Reaction time and accuracy were recorded without providing feedback to the participant.

### 2.3 EEG recordings

EEGs were recorded with a sample rate of 512 Hz and cutoff frequencies of 0.1–50 Hz via 19 Ag/AgCl cup electrodes fixed to the scalp with electrolyte gel. Electrodes were placed at positions with impedances <5 kOhm. These included the 19 standard electrodes of the 10–20 system (Penfield and Jasper, 1954) and Cz as the recording reference. The Schwarzer EEG-29T recording system with Coherence version 6.1.3.417 application software (Natus Europe GmbH) was used.

### 2.4 EEG data preprocessing and data processing

EEG data were analyzed offline using Matlab (The MathWorks, Inc.)-based EEG analysis software products, EEGLAB^2^ (Delorme and Makeig, 2004), with some custom processing scripts.

Data prepossessing and further EEG analyses were performed with EEGLAB. Electrodes was re-refenced into average reference. The 50 Hz line noise in the EEG signals was rejected using bandpass filters with a value of 50 Hz; after that, a cutoff frequency of 0.1–50 Hz was applied. Then, the EEG signals with performance errors or remaining artifacts exceeding ± 120 μV in any channel, along with eye-blink artifacts, were rejected from the data before processing using the ICA procedure (EEGLAB tutorial, 2026).

The study included the aforementioned set of ROIs, representing memory encoding and verbal information processing, specifically F7 and F3, which are associated with Broca’s area (left inferior frontal/frontotemporal and left dorsolateral prefrontal cortex, respectively). Cumulative event data were then calculated for each experimental encoding or decoding step. Decoding events were calculated separately for each answer type (Hit, Miss, Correct Rejection, and False Alarm).

Tasks for the Time-Frequency (TF) analysis of different list lengths were formed by combining experimental sessions of 5, 7, 10, 24, 36, 50, 80, 140, and 225 words (9 tasks). TF analysis for event-related spectral perturbation (ERSP) was performed over the time window of −200 to 1,500 ms relative to the stimulus presentation. This was done using the ERPLAB Study pipeline for each group of stimuli during the encoding phase. The TF analysis was calculated with the following parameters: cycles 3 0.8, nfreqs 100, ntimesout 200.

The spectral EEG signal was analyzed for commonly used wave frequencies by calculating their spectral power. This was performed using the EEGLAB spectopo function with a frequency parameter of 1–48 Hz for each event-related time epoch.

For each serial number i up to the maximum list length *N*, the average spectral power *p_i_*, (*i* = 1,2,3,…,*N*) was calculated for the sample of observation on a particular number of N. Then, the average cumulative spectral power $P_{n_{i}}$ was calculated within the interval of a particular list length $P_{n_{i}},(n_{i}=1,2,3\ldots ,i):P_{n_{i}}=(\sum_{i=1}^{n_{i}}p_{i})/n_{i}$ , for the selected range of EEG waves.

### 2.5 Statistical analysis

An analysis of variance (ANOVA) with list length as the within-subject factor was calculated for the top-level analysis of the TF with False Discovery Rate (FDR) correction and Spectral analysis. These calculations were performed in EEGLAB using the STUDY functions (EEGLAB tutorial) with 23 participants, 9 tasks of list lengths, EEGLAB statistics, a statistical threshold level of 0.05, and permutation statistics (*n* = 1,000) for the parametric model, ensuring the robustness of the inferences. They compared TF in different list lengths within the windows of 0–1,500 ms for five experimental encoding or decoding event types. Spectral power within the classical EEG wave bands (1–48 Hz) was computed by comparing the current point’s spectral power with the averaged spectral power derived from the preceding points in the series. For all tests, the *p*-level <0.05 was considered significant.

## 3 Results

### 3.1 TF analysis

**Figure 2:**
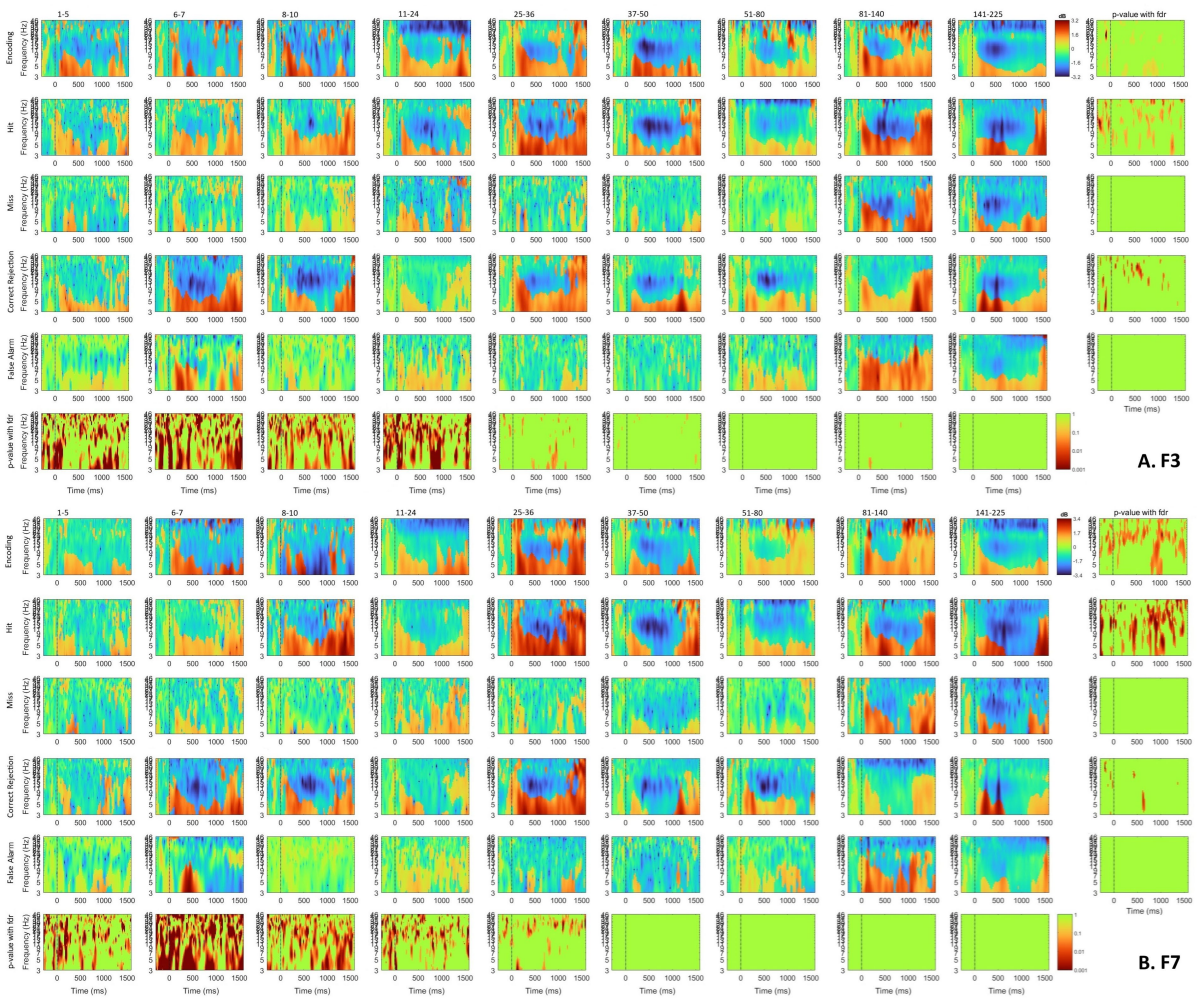
Time-frequency plot of the selected list length position during encoding and recognition events, along with the ANOVA results. **(A)** F3 electrode, **(B)** F7 electrode.

Figure 2 presents data from two regions of interest (ROIs) under the F7 and F3 electrodes, which correspond to Broca’s area. Two activity patterns were observed in both channels. The first pattern reflects the similarity between correct responses (Hits and Correct Rejections) and the encoding stage. The second pattern is observed during error events (Misses and False Alarms). At the same time, these trends were not pronounced when the list length exceeded 80 words, and all responses had patterns closer to the encoding phase.

In both electrodes, high spectral power was observed in the theta and delta bands (50–500 ms) during encoding and for correct answer cases. Conversely, low spectral power was found in the alpha and beta bands (300–1,000 ms). In some instances, beta activity appeared in the 300–400 ms time window alongside alpha activity. The gamma band exhibited high variability in activation patterns across all observed cases.

The ANOVA showed significant differences in the shortest lists (under 37 words) for all response types. Among list lengths, differences were observed within the encoding and correct recognition events (Hits and Correct Rejections; see Figures 2A,B), while no significant changes were found in error recognition events (Misses and False Alarms).

### 3.2 Spectral analysis

**Figure 3:**
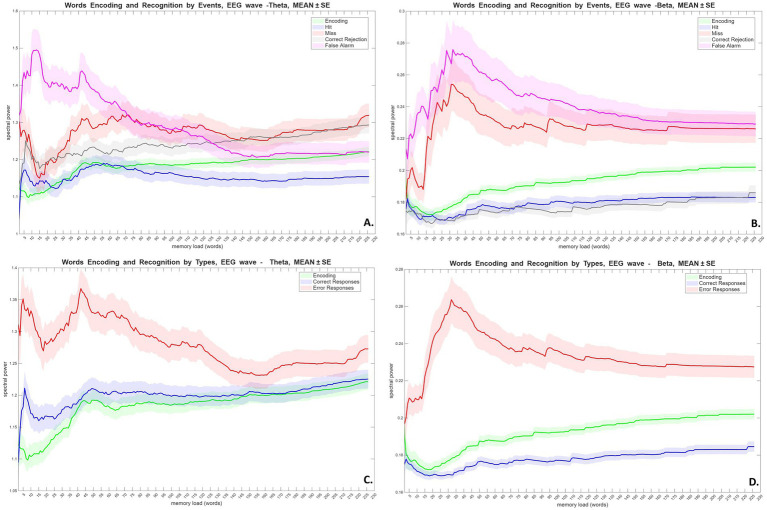
Theta and beta wave spectral power during encoding and recognition events in a serial list position. **(A)** Theta wave with all experimental events, **(B)** beta wave with all experimental events, **(C)** theta wave—encoding and correct or error event types, **(D)** beta wave—encoding and correct or error event types.

The spectral analysis of EEG waves was performed using the cumulative average of the F3 and F7 electrodes’ spectral power. Two different visual patterns of memory processes were observed (see Figure 3). The first pattern involved error recognition events (Misses and False Alarms). This pattern required higher spectral power than the second pattern, which involved encoding and correct recognition events (Hits and Correct Rejections). These patterns are better identifiable in the beta band than in the theta band waves.

Another finding is the presence of step-like, non-linear spectral power lines under all observed events, especially during the first 120 list positions. However, a greater change in the absolute value of a particular spectral power is required for a significant effect on the cumulative average power with longer list lengths. Therefore, decreasing step-like changes (e.g., during encoding or Hits) did not confirm the disappearance of a possible underlying process at longer list lengths.

Differences between correct recognition and error events become clearly visible when they are grouped, and in the beta band, spectral power variability is particularly evident.

## 4 Discussion

In general, the study revealed characteristics of neural activity in Broca’s area during the encoding and recognition of verbal information under variable memory loads, ranging from short (2–36 words) to very long (225 words) lists. The findings revealed distinct patterns for correct and incorrect responses. Across ROIs, the oscillatory patterns of information processing in Broca’s area were similar for encoding and correct recognition responses (Hits and Correct Rejections). This is consistent with studies emphasizing the role of this area in verbal working memory (e.g., Chai et al., 2018). In contrast, error responses (Misses and False Alarms) showed a distinct pattern, characterized by higher power in the theta and beta frequency bands. This points to a possible overconsumption of energy during incorrect search and decision-making processes. However, the differences between correct and error responses diminished as list length increased.

These observed changes revive the discussion on LTWM (Ericsson and Kintsch, 1995) and its limitations (Jensen et al., 2002; Sauseng et al., 2009; Staufenbiel et al., 2014). TF analysis demonstrated that the initially observed differences in oscillatory patterns between correct and incorrect reactions decreased, indicating changes in memory processes in the region beyond 80 words. At the same time, spectral power analysis indicated gradual convergence of incorrect reaction neural activity with correct reaction neural activity in the theta and beta bands. This convergence occurred from maximum divergence at 30–50 words to convergence at approximately 150–170 words. The local or global maximums of spectral power in the 30–50 word interval were found to be in line with findings that the 30–59 word region significantly differs in episodic memory encoding trends from shorter and longer lists (Veliks et al., 2025).

Another notable finding was the presence of step-like changes in cumulative spectral power. Accounting for the limited information processing capacity resulting from the restricted involvement of groups of neocortical neurons (e.g., Hill and Dunbar, 2003), it was possible to assume a stepwise mechanism of neuronal involvement in memory processing. To support the testing of this assumption in further studies, we propose delineating an analogy between the quantitative characteristics of list lengths and the number of individuals in groups of different closeness. The observed local and absolute maxima of the spectral power of theta and beta frequency bands within the 30–50 word range concurred with the 50-individual size limit for a social group of affinity (Sutcliffe et al., 2012). The convergence interval for the power of neural oscillations under correct and incorrect responses (150–170 words) was close to the 150–153.5 range, mentioned by Hill and Dunbar (2003) as the limit on the number of individuals in a personal social network. Thus, our study points to some convergence between non-behavioral and behavioral data from different scientific domains. The mentioned volumes of episodic memory corresponded to the maximum size of an affinity group and an active personal social network (Sutcliffe et al., 2012), which are associated with different qualities and volumes of information stored in an individual’s memory regarding closer (50) and less close (150) individuals.

Unlike Weiss and Rappelsberger (2000), who noted an increase in lower beta power during word encoding, our data refined this result and showed more complex power dynamics as a function of the list length. This quasi-linear increase was generally noted after 20 encoded items (Figure 3) and was preceded by a decrease or relative plateau in power. The dynamics of theta power are also complex. The observed increase in theta wave power appeared to play a role in encoding and processing verbal information across the brain regions involved in this task.

A comparison of spectral patterns beyond 450–500 ms (Figure 2) revealed a systematic desynchronization of waves in the lower beta band (12–20 Hz) at different list lengths during encoding and successful word recognition, which, according to Hanslmayr et al. (2009, 2016), is due to the processes of their deep semantic coding. This coding is not typical for recognition errors (misses and false alarms). Interestingly, in the case of misses, such desynchronization was clearly expressed for lists longer than 80 items. This may indicate more intensive semantic processing in cases of search failures when recognizing the encoded target words of such lists, in addition to a difference in the occurrence mechanisms of alternative types of errors.

The observed differences in the window after 450 ms in ROI F3 and F7 (see Figure 3) pointed to a more active involvement of the more lateralized (F7) left-hemispheric region during encoding under different conditions (Wiesman et al., 2021). These also indicated the intensive involvement of working memory regions in processing the most extended sets of stimuli (Jeneson and Squire, 2012).

The revealing of trends also points to significant limitations in the current study. Trends under the longest list lengths (over 200 words) were calculated based on a limited number of observations. Caution is required when interpreting data from this interval. Focusing on the beta and theta bands limited the inclusion of other waves in the analysis. Moreover, a limited number of ROIs (F3 and F7) showed trends that were relatively isolated from other oscillatory activities. The gender composition of the sample was homogeneous and unchangeable because of the analysis of existing data. This constituted another limitation, which should be balanced in further studies.

While providing no direct clinical insights, the present study reveals some opportunities for use in individual assessments. Differences in power trends during encoding and during correct and incorrect recognition provide a basis for comparing individual results with observed patterns. This is especially relevant for incorrect functioning under tasks with short- and medium-length word lists, as more energy-consuming patterns were observed.

In summary, the study shows differences in oscillatory patterns during verbal encoding and recognition across different list lengths. Correct responses showed consistency in theta and beta activity, with relatively low spectral power, whereas incorrect responses showed signs of inefficient processing (power overuse). These differences were the most pronounced at 30–50 items, and some convergence of correct and incorrect activity patterns was observed around 150–170 items. Stepwise shifts in cumulative spectral power require more detailed investigation of the recruitment of neural resources as list length increases. In addition, a systematic lower-beta desynchronization during successful processing adds to the findings, which could be a sign of Broca’s area’s adaptation to memory load.

## Data Availability

The raw data supporting the conclusions of this article will be made available by the authors, without undue reservation.

## Footnotes

1
http://psychtoolbox.org/

2
http://sccn.ucsd.edu

## References

[ref1] Alexandrov A. Boricheva D. Pulvermüller F. Shtyrov Y. Zhang L. I. (2011). Strength of word-specific neural memory traces assessed electrophysiologically. PLoS One 6:e22999. doi: 10.1371/journal.pone.0022999, 21853063 PMC3154264

[ref2] Annis J. Lenes J. G. Westfall H. A. Criss A. H. Malmberg K. J. (2015). The list-length effect does not discriminate between models of recognition memory. J. Mem. Lang. 85, 27–41. doi: 10.1016/j.jml.2015.06.001

[ref3] Baddeley A. (2012). Working memory: theories, models, and controversies. Annu. Rev. Psychol. 63, 1–29. doi: 10.1146/annurev-psych-120710-100422, 21961947

[ref4] Bulut T. (2023). Domain-general and domain-specific functional networks of Broca’s area underlying language processing. Brain Behav. 13:e3046. doi: 10.1002/brb3.3046, 37132333 PMC10338813

[ref5] Burrows D. Okada R. (1975). Memory retrieval from long and short lists. Science 188, 1031–1033. doi: 10.1126/science.188.4192.1031, 17759685

[ref6] Cary M. Reder L. M. (2003). A dual-process account of the list-length and strength-based mirror effects in recognition. J. Mem. Lang. 49, 231–248. doi: 10.1016/S0749-596X(03)00061-5

[ref7] Cavanah P. J. Fiebelkorn I. C. (2026). A shared theta-rhythmic process for selective sampling of environmental information and internally stored information. J. Neurosci. 46:e1560252026. doi: 10.1523/JNEUROSCI.1560-25.2026, 41702719 PMC12981286

[ref8] Chai W. J. Abd Hamid A. I. Abdullah J. M. (2018). Working memory from the psychological and neurosciences perspectives: a review. Front. Psychol. 9:401. doi: 10.3389/fpsyg.2018.00401, 29636715 PMC5881171

[ref9] Chmiel J. Kurpas D. (2026). Mapping executive function performance based on resting-state EEG in healthy individuals: a systematic and mechanistic review. J. Clin. Med. 15:1306. doi: 10.3390/jcm15031306, 41682987 PMC12898202

[ref10] Delorme A. Makeig S. (2004). EEGLAB: an open source toolbox for analysis of single-trial EEG dynamics. J. Neurosci. Methods 134, 9–21. doi: 10.1016/j.jneumeth.2003.10.009, 15102499

[ref11] Dennis S. Humphreys M. S. (2001). A context noise model of episodic word recognition. Psychol. Rev. 108, 452–478. doi: 10.1037/0033-295x.108.2.452, 11381837

[ref12] Dennis S. Lee M. D. Kinnell A. (2008). Bayesian analysis of recognition memory: the case of the list-length effect. J. Mem. Lang. 59, 361–376. doi: 10.1016/j.jml.2008.06.007

[ref13] EEGLAB tutorial (2026). EEGLAB tutorial. Available online at: https://eeglab.org/tutorials/06_RejectArtifacts/RunICA.html (Accessed April 16, 2026).

[ref14] Ericsson K. A. Kintsch W. (1995). Long-term working memory. Psychol. Rev. 102, 211–245. doi: 10.1037/0033-295X.102.2.211 7740089

[ref15] Graboi D. Lisman J. (2003). Recognition by top-down and bottom-up processing in cortex: the control of selective attention. J. Neurophysiol. 90, 798–810. doi: 10.1152/jn.00777.2002, 12702712

[ref16] Günseli E. Foster J. J. Sutterer D. W. Todorova L. Vogel E. K. Awh E. (2026). Encoded and updated spatial work in g memories share a common representational format in alpha activity. Cell Press J. 27:108963. doi: 10.1016/j.isci.2024.108963, 38333713 PMC10850742

[ref17] Hanslmayr S. Spitzer B. Baeuml K. H. (2009). Brain oscillations dissociate between semantic and non-semantic encoding of episodic memories. Cereb. Cortex 19, 1631–1640. doi: 10.1093/cercor/bhn197, 19001457

[ref18] Hanslmayr S. Staresina B. P. Bowman H. (2016). Oscillations and episodic memory: addressing the synchronization/desynchronization conundrum. Trends Neurosci. 39, 16–25. doi: 10.1016/j.tins.2015.11.004, 26763659 PMC4819444

[ref19] Hill R. A. Dunbar R. I. M. (2003). Social network size in humans. Hum. Nat. 14:1.53-72. doi: 10.1007/s12110-003-1016-y, 26189988

[ref20] Jeneson A. Squire L. R. (2012). Working memory, long-term memory, and medial temporal lobe function. Learn. Mem. 19, 15–25. doi: 10.1101/lm.024018.111, 22180053 PMC3246590

[ref21] Jensen O. Gelfand J. Kounios J. Lisman J. E. (2002). Oscillations in the alpha band (9–12 Hz) increase with memory load during retention in a short-term memory task. Cereb. Cortex 12, 877–882. doi: 10.1093/cercor/12.8.877, 12122036

[ref22] Kavanaugh B. C. Vigne M. M. Thorpe R. Legere C. Acuff W. L. Vaughan N. . (2026). The association between oscillatory burst features and human working memory accuracy unavailable. J. Cogn. Neurosci. 38, 281–298. doi: 10.1162/JOCN.a.87, 40811625 PMC12927161

[ref23] Kleiner M. Brainard D. Pelli D. Ingling A. Murray R. Broussard C. (2007). What’s new in Psychtoolbox-3? Perception 36, 1–16. doi: 10.1177/03010066070360S101

[ref24] Murdock B. B. Kahana M. J. (1993). List-strength and list-length effects: reply to Shiffrin, Ratcliff, Murnane, and M. Cary, L.M. Reder. J. Mem. Lang. 49, 231–248. 10.1037/0278-7393.19.6.1445 8270891

[ref25] Murnane K. Shiffrin R. (1991). Interference and the representation of events in memory. J. Exp. Psychol. Learn. Mem. Cogn. 17, 855–874. doi: 10.1037/0278-7393.17.5.855, 1834768

[ref26] Ohrt D. D. Gronlund S. D. (1999). “List-length effect and continuous memory: confounds and solutions,” in On Human Memory: Evolution, Progress, and Reflections on the 30th Anniversary of the Atkinson-Shiffrin Model, ed. Izawa C. (Mahwah: Lawrence Erlbaum Associates), 105–125.

[ref27] Omelyusik V. Davis T. S. Nair S. S. Noudoost B. Hackett P. D. Smith E. H. . (2026). Frontotemporal bursting supports human working memory. NeuroImage 327:121718. doi: 10.1016/j.neuroimage.2026.121718, 41539468 PMC13143355

[ref28] Penfield W. Jasper H. H. (1954). Epilepsy and the Functional Anatomy of the Human Brain. Boston: Little, Brown & Co.

[ref29] Peters J. O. Steiger T. K. Sobczak A. Bunzeck N. (2022). Set size of information in long-term memory similarly modulates retrieval dynamics in young and older adults. Front. Psychol. 13:817929. doi: 10.3389/fpsyg.2022.817929, 35310276 PMC8924055

[ref30] Rao A. M. DeHaan R. D. Kahana M. J. (2025). Synchronous theta networks characterize successful memory retrieval. J. Neurosci. 45:e1332242025. doi: 10.1523/JNEUROSCI.1332-24.2025, 40032520 PMC12005240

[ref31] Ratcliff R. Murdock B. B. Jr. (1976). Retrieval processes in recognition memory. Psychol. Rev. 83, 190–214. doi: 10.1037/0033-295X.83.3.190

[ref32] Sauseng P. Klimesch W. Heise K. F. Gruber W. R. Holz E. Karim A. A. (2009). Brain oscillatory substrates of visual short-term memory capacity. Curr. Biol. 19, 1846–1852. doi: 10.1016/j.cub.2009.08.062, 19913428

[ref33] Shtyrov Y. Pulvermüller F. (2007). Language in the mismatch negativity design: motivations, benefits, and prospects. J. Psychophysiol. 21, 176–187. doi: 10.1027/0269-8803.21.34.176

[ref34] Solomon E. A. Kragel J. E. Sperling M. R. Sharan A. Worrell G. Kucewicz M. . (2017). Widespread theta synchrony and high-frequency desynchronization underlies enhanced cognition. Nat. Commun. 8:1704. doi: 10.1038/s41467-017-01763-2, 29167419 PMC5700170

[ref35] Staufenbiel S. M. Brouwer A. M. Keizer A. W. van Wouwe N. C. (2014). Effect of beta and gamma neurofeedback on memory and intelligence in the elderly. Biol. Psychol. 95, 74–85. doi: 10.1016/j.biopsycho.2013.05.020, 23751914

[ref36] Sternberg S. (1966). High-speed scanning in human memory. Science 153, 652–654. doi: 10.1126/science.153.3736.652, 5939936

[ref37] Sutcliffe A. Dunbar R. Binder J. Arrow H. (2012). Relationships and the social brain: integrating psychological and evolutionary perspectives. Br. J. Psychol. 103, 149–168. doi: 10.1111/j.2044-8295.2011.02061.x, 22506741

[ref38] Tremblay P. Dick A. (2016). Broca and Wernicke are dead, or moving past the classic model of language neurobiology. Brain Lang. 162, 60–71. doi: 10.1016/j.bandl.2016.08.004, 27584714

[ref39] Tromp D. Dufour A. Lithfous S. Pebayle T. Després O. (2015). Episodic memory in normal aging and Alzheimer disease: insights from imaging and behavioral studies. Ageing Res. Rev. 24, 232–262. doi: 10.1016/j.arr.2015.08.006, 26318058

[ref40] Turner B. M. Dennis S. Van Zandt T. (2013). Likelihood-free Bayesian analysis of memory models. Psychol. Rev. 120, 667–678. doi: 10.1037/a0032458, 23586446 PMC4140406

[ref41] Veliks V. Kolesovs A. Porozovs J. Igonins D. (2025). Effects of word list length during episodic memory encoding observation by the event-related potential and time-frequency. Front. Hum. Neurosci. 19:1542289. doi: 10.3389/fnhum.2025.1542289, 40331013 PMC12052787

[ref42] Weiss S. Rappelsberger P. (2000). Long-range EEG synchronization during word encoding correlates with successful memory performance. Brain Res. Cogn. Brain Res. 9, 299–312. doi: 10.1016/s0926-6410(00)00011-2, 10808141

[ref43] Wiesman A. I. Christopher-Hayes N. J. Wilson T. W. (2021). Stairway to memory: left-hemispheric alpha dynamics index the progressive loading of items into a short-term store. NeuroImage 235:118024. doi: 10.1016/J.NEUROIMAGE.2021.118024, 33836267 PMC8354033

[ref44] Wong S. T. S. Goghari V. M. Sanford N. Lim R. Clark C. Metzak P. D. . (2020). Functional brain networks involved in lexical decision. Brain Cogn. 138:103631. doi: 10.1016/j.bandc.2019.103631, 31835145

[ref45] Wynn S. C. Townsend C. D. Nyhus E. (2024). The role of theta and gamma oscillations in item memory, source memory, and memory confidence. Psychophysiology 61:e14602. doi: 10.1111/psyp.14602, 38715221 PMC11330366

